# Mechanistic insights into neutrophil involvement in liver transplant ischemia-reperfusion injury and rejection

**DOI:** 10.3389/fimmu.2026.1754165

**Published:** 2026-01-22

**Authors:** Zhipeng She, Hailun Cai, Xinqiang Li, Jinhui Chen, Ying Chen, Jinzhen Cai, Bin Wu

**Affiliations:** 1 Organ Transplant Center, Fujian Medical University Union Hospital, Fuzhou, China; 2 Organ Transplantation Center, Affiliated Hospital of Qingdao University, Qingdao, China

**Keywords:** immune rejection, ischemia-reperfusion injury, liver transplantation, neutrophils, postoperative complications

## Abstract

Ischemia-reperfusion injury (IRI) and subsequent rejection remain the paramount pathological barriers to long-term graft survival following liver transplantation. Traditionally viewed as mere ‘first responders’ in the acute inflammation of IRI, neutrophils are now recognized, based on recent advances, as pivotal regulators that bridge innate and adaptive immunity throughout the entire post-transplant course. This review aims to systematically delineate the dual pathological mechanisms of neutrophils in both IRI and rejection post-LT. During the initial phase of IRI, we focus on the robust activation of neutrophils, driven by damage-associated molecular patterns (DAMPs), with a particular emphasis on the formation of neutrophil extracellular traps (NETs). NETs act not only as key effectors causing sinusoidal microcirculatory dysfunction and direct hepatocellular injury, but their released histones and proteases also serve as potent danger signals, amplifying the local inflammatory cascade. The central thesis of this review is that the inflammatory microenvironment, orchestrated by neutrophils during IRI, provides the essential immunological substrate for subsequent rejection. We delve into the mechanisms by which neutrophils bridge to adaptive immunity: NETs serve as a scaffold for autoantigens, activating B cells and promoting the production of donor-specific antibodies (DSA), thereby driving antibody-mediated rejection (AMR). Concurrently, chemokines released by neutrophils efficiently recruit effector T cells and, through interactions with antigen-presenting cells, exacerbate cell-mediated rejection (CMR). Finally, we prospect future therapeutic directions, emphasizing that targeting common pathways within this ‘injury-immunity’ axis—such as inhibiting DAMP release, blocking NET formation, or employing pro-resolving mediators to actively terminate inflammation—represents a pivotal strategy to break the vicious cycle of IRI and rejection and achieve long-term immune tolerance.

## 1 Introduction

Liver transplantation is an effective treatment for end-stage liver diseases. However, during the transplantation process, there are numerous risk factors and complications that can lead to various adverse outcomes for patients (1). In liver transplantation, ischemia-reperfusion injury (IRI) and immune rejection are major complications. Although various strategies exist to mitigate their effects, they have not been entirely resolved, and a complete solution has yet to be found. IRI is a multifaceted pathophysiological process wherein the sequential insults of warm and cold ischemia and reperfusion synergistically trigger a deleterious cascade—encompassing oxidative stress, reactive oxygen species (ROS) generation, adenosine triphosphate (ATP) depletion, mitochondrial impairment, microcirculatory disturbances, immune cell infiltration, and pro-inflammatory mediator release—that culminates in cellular damage and necrosis (2, 3). The occurrence of IRI may cause hepatocytes damage, microcirculation disorders, and biliary complications, and is closely related to the occurrence of transplantation failure (4). The core mechanism of liver transplant rejection is the recipient’s immune system’s recognition of and attack on the donor allograft as “non-self,” which is a primary cause of graft dysfunction. Existing research indicates that the innate immune response, initiated by IRI, plays a dual role in the immunological cascade of rejection. It acts as the foundational trigger that both initiates the response and subsequently magnifies its magnitude through the activation of inflammatory cells. Therefore, it is crucial to investigate IRI and rejection as an integrated entity rather than as separate phenomena (2, 5, 6).

Neutrophils, as the initial infiltrating white blood cell subset following transplantation, represent a critical cellular component implicated in the pathogenesis of IRI and rejection responses. Accumulating evidence from recent studies highlights the pivotal role of the immune system in mediating the progression and development of IRI and rejection reactions (7, 8). In this review, we have elaborated on the role of neutrophils in the IRI and rejection reaction that occurs during liver transplantation.

## 2 The biological characteristics of neutrophils

### 2.1 General characteristics of neutrophils

Neutrophils are produced by hematopoietic stem cells in the bone marrow via the myeloid differentiation pathway and constitute the primary immune cells in blood circulation (9, 10). Under various microenvironmental stimuli, neutrophils predominantly polarize into N1 and N2 subtypes (11), N1-type neutrophils release ROS, elastase, myeloperoxidase (MPO), and other substances to promote inflammation. They also enhance pro-inflammatory effects by augmenting the Th1/Th17 immune response (12); Furthermore, proteases secreted by N1-type neutrophils can degrade the extracellular matrix (ECM) and impede tissue repair (13). In contrast, N2-type neutrophils secrete anti-inflammatory cytokines such as interleukin-10 (IL-10) and transforming growth factor-beta (TGF-*β*) to suppress inflammatory responses. Additionally, they produce growth factors like vascular endothelial growth factor (VEGF), hepatocyte growth factor (HGF), and matrix metalloproteinase-9 (MMP-9) to facilitate angiogenesis and tissue remodeling (14–16). During infections, neutrophils respond rapidly within 1–2 hours and recruit additional immune cells to the infection site by secreting chemokines (17). Researchers have discovered that the immune regulatory ability of neutrophils is related to the various mediators they secrete, including cytokines and extracellular vesicles (EVs) containing components such as microRNAs (miRNAs) (18–20). Neutrophils not only release antibacterial substances, including antimicrobial peptides, highly active serine proteases, and ROS, but also eliminate pathogenic microorganisms through phagocytosis (21) (**Table 1**).

**Table 1:** Summary of the receptors of neutrophils during liver transplantation process.

Receptor	Receptor type	Main ligand/activator	Subcellular localization	Reference
CXCR1/CXCR2	GPCR	CXCL1; CXCL2; CXCL5; CXCL8 (IL-8)	Cytomembrane	(22–24)
TLR2	TLR, PRR	DAMPs	Cytomembrane	(25)
TLR4	TLR, PRR	LPS; HMGB1; HSP70	Cytomembrane	(26, 27)
Integrins (CD11b/CD18, Mac-1)	Adhesion molecule	ICAM-1; Fibrinogen	Cytomembrane	(28, 29)
RAGE	IgSF receptor	HMGB1; S100 protein;	Cytomembrane	(30)
Fc*γ* Receptors (CD64, CD32, CD16)	FcR	IgG	Cytomembrane	(31, 32)
CXCR3	GPCR	CXCL9; CXCL10; CXCL11	Cytomembrane	(33)
Glucocorticoid Receptor(GR)	Nuclear receptor	Glucocorticoid	Nucleus (Site of Action)	(34, 35)
Peroxisome Proliferator-Activated Receptor Gamma (PPAR*γ*)	Nuclear receptor	15d-PGJ2 (endogenous); Rosiglitazone	Nucleus (Site of Action)	(36)
Estrogen Receptor(ER)	Nuclear receptor	Estradiol	Nucleus (Site of Action)	(37)

### 2.2 Characteristics of neutrophils during IRI and rejection reaction

During LIRI, neutrophils exhibit profound heterogeneity and plasticity. While frequently dichotomized into N1 and N2 subsets, this paradigm serves as a dynamic, context-dependent framework rather than a rigid classification predicated on definitive surface markers (38). This distinction is vital, as precise phenotypic signatures for neutrophil subsets in liver transplantation remain to be fully established. Ultimately, the functional trajectory of these cells, governed by complex microenvironmental signaling, dictates graft prognosis.

Neutrophil differentiation initiates with signal activation and cascade recruitment. Within the transplant microenvironment, ischemia-induced hypoxia subsequently inhibits prolyl hydroxylases (PHDs), stabilizing hypoxia-inducible factor-1*α* (HIF-1*α*). This stabilization not only prolongs neutrophil survival by upregulating anti-apoptotic proteins (e.g., Mcl-1) and inhibiting caspases but simultaneously drives neutrophil reprogramming toward an immunoregulatory phenotype through glycolytic remodeling and the induction of immunosuppressive molecules. This reprogramming represents a systematic and reversible phenotypic remodeling process, characterized by comprehensive alterations in metabolic modalities (e.g., glycolysis and oxidative phosphorylation), surface markers, secretomes, effector functions (including T cell modulation and NET formation), survival kinetics, and subset composition, Consequently, neutrophils undergo a functional transition from classic pro-inflammatory/bactericidal states toward heterogeneous phenotypes associated with immunosuppression, tissue repair, or tumor promotion (39–41).

Necrotic hepatocytes release damage-associated molecular patterns (DAMPs)—HMGB1 (activating Toll-like receptor 4/MyD88/NF-*κ*B), mitochondrial DNA (activating Toll-like receptor 9/cGAS-STING), and ATP (activating P2X7/NLRP3)—that potently activate neutrophils (42–45). This signaling milieu drives neutrophil differentiation into a pro-inflammatory/cytotoxic subset. Characterized by high CD11b, CD62L shedding, and potent oxidative bursts, this subset inflicts damage primarily through ROS/MPO release and neutrophil extracellular traps (NETs) formation (46). NETs cause microcirculatory dysfunction, and their components (e.g., citrullinated histones, MPO-DNA complexes) act as secondary DAMPs. These DAMPs are recognized by dendritic cells (DCs) via Toll-like receptor 4(TLR4), thereby enhancing their own reactivity (47, 48). Exposure to NETs induced the secretion of BAFF by neutrophils, BAFF is closely related to antibody-mediated rejection reactions in organ transplantation (49, 50). Pro-inflammatory neutrophils attract effector T cells through the C-X-C motif chemokine ligand9/10(CXCL9/10) signaling pathway, thereby triggering a T-cell-mediated rejection reaction (33, 51). Consequently, it impacts the rejection reaction.

The main understanding of neutrophils in IRI is their pro-inflammatory effect. Currently, the research on their anti-inflammatory role is not complete, many theories are still at the stage of hypothesis. Relevant research reports indicate that during the period of inflammation subsiding, the N2 type neutrophils also play a certain role. This sub - population exhibits high expression characteristics of PD - L1 and Arg - 1. It induces T cells to enter an exhausted state through the PD - L1/PD - 1 pathway, thereby suppressing the immune response (52–54). The resolution of inflammation is partly attributed to specialized pro-resolving mediators (SPMs), a novel class of endogenous lipids that play a pivotal role in regulating neutrophil functions. Emerging evidence indicates that beyond their canonical roles in inhibiting recruitment, limiting activation, and promoting apoptosis, SPMs also reprogram neutrophils into a pro-repair phenotype (55, 56). This phenotype is characterized by the secretion of tissue-reparative factors such as VEGF and TGF-*β* (57). The neutrophil-orchestrated inflammatory milieu during IRI creates the immunological soil for subsequent rejection.

Ultimately, neutrophils undergo a distinct form of programmed cell death, known as NETosis, to extrude NETs during ischemia-reperfusion. These structures are essentially a meshwork of DNA fibers, histones, and cytotoxic granular proteins. NETs are widely recognized as key mediators of tissue damage in LIRI (58–61). Alternatively, the sequestration of DAMPs by NETs has been proposed as a tissue-protective mechanism that mitigates excessive inflammation (62). Although direct experimental confirmation of a protective role in liver IRI is still evolving, studies in other transplant models, such as the kidney, have demonstrated that NETs can indeed limit IRI severity (63, 64).

Therefore, the dynamic interplay between these subsets, not a single neutrophil population, dictates graft fate—injury, repair, or tolerance. This makes targeting specific subset functions—such as inhibiting NETosis or enhancing pro-resolving phenotypes—a compelling therapeutic strategy.

## 3 The activities of neutrophils

### 3.1 Activation and chemotaxis of neutrophils

The discovery of TLRs as detectors of pathogen-associated molecular patterns (PAMPs), alongside Matzinger’s ‘danger model’ of endogenous DAMPs, has revolutionized our understanding of immune activation (65, 66). During the ischemic phase of LIRI, necrotic hepatocytes release a barrage of DAMPs, including HMGB1, ATP, and mitochondrial DNA (67). While Toll-like receptors (TLRs) evolved primarily to recognize exogenous PAMPs for infection defense, they cross-recognize these endogenous DAMPs via molecular mimicry in the transplant setting, thereby bridging sterile injury to the immune response (68).

HMGB1, a key initiator of inflammation, initiates inflammatory signaling through multiple pathways (69). Firstly, it activates the cell membrane receptor Toll-like receptor 2 (TLR2). Via the cytoplasmic adaptor protein MyD88, this activation leads to the recruitment of the MAPK and IKK signaling cascades. Consequently, the transcription factors NF-*κ*B and Activator Protein-1 (AP-1) are induced, mediating cytokine transcription (70). Concurrently, endogenous HMGB1 also engages the TLR4 receptor. This receptor signals not only through the MyD88-dependent pathway to recruit MAPK but also via the TRIF-dependent pathway to activate both MAPK and IKK, culminating in the mediation of cytokine transcription (25, 42). In addition to TLRs, endogenous HMGB1 activates the Receptor for Advanced Glycation End-products (RAGE), a transmembrane protein of the immunoglobulin (Ig) superfamily. Through the PI3K/AKT and MAPK pathways, RAGE activation also leads to the induction of NF-*κ*B and AP-1, thereby promoting the inflammatory response (30). Relevant experiments indicate that PI3K/Akt activation can mitigate LIRI in specific contexts via increased microenvironmental nitric oxide generation, underscoring the pathway’s context-dependent and cell-specific effects (71, 72). Beyond HMGB1, other DAMPs like RNA and DNA engage TLR3 and TLR9, respectively, with TLR9 signaling playing a predominant role in activating neutrophils to produce pro-inflammatory cytokines and chemokines during LIRI (44). Inhibition of TLR9 suppresses the expression of peptidyl-arginine deiminase-4 (PAD4) and Rac2. This is mechanistically significant, as the formation of NETs is dependent on both histone citrullination catalyzed by PAD4 and the production of ROS by NADPH oxidase (73). Therefore, targeting TLR9 presents a promising therapeutic strategy for the treatment of ischemia-reperfusion injury.

During the reperfusion phase, the surge in ROS and inflammatory mediator from resident immune cells further activates neutrophils. Kupffer cells release pro-inflammatory cytokines such as IL-6, IL-1*β*, and TNF-*α*, while damaged hepatocytes and Kupffer cells secrete chemokines including IL-1, CXCL2, and CXCL8. These chemokines bind to C-X-C motif chemokine receptor 1/2(CXCR1/2) on neutrophils, establishing a chemotactic gradient that guides their migration towards the injured liver (22, 74). This recruitment is tightly regulated by several critical signaling pathways. Carcinoembryonic antigen-related cell adhesion molecule 1 (CEACAM1), particularly the CC1-L isoform, acts as a crucial checkpoint that inhibits neutrophil activation by suppressing the ASK1/p-p38 pathway, thereby mitigating IRI (75, 76).

**Figure 1:**
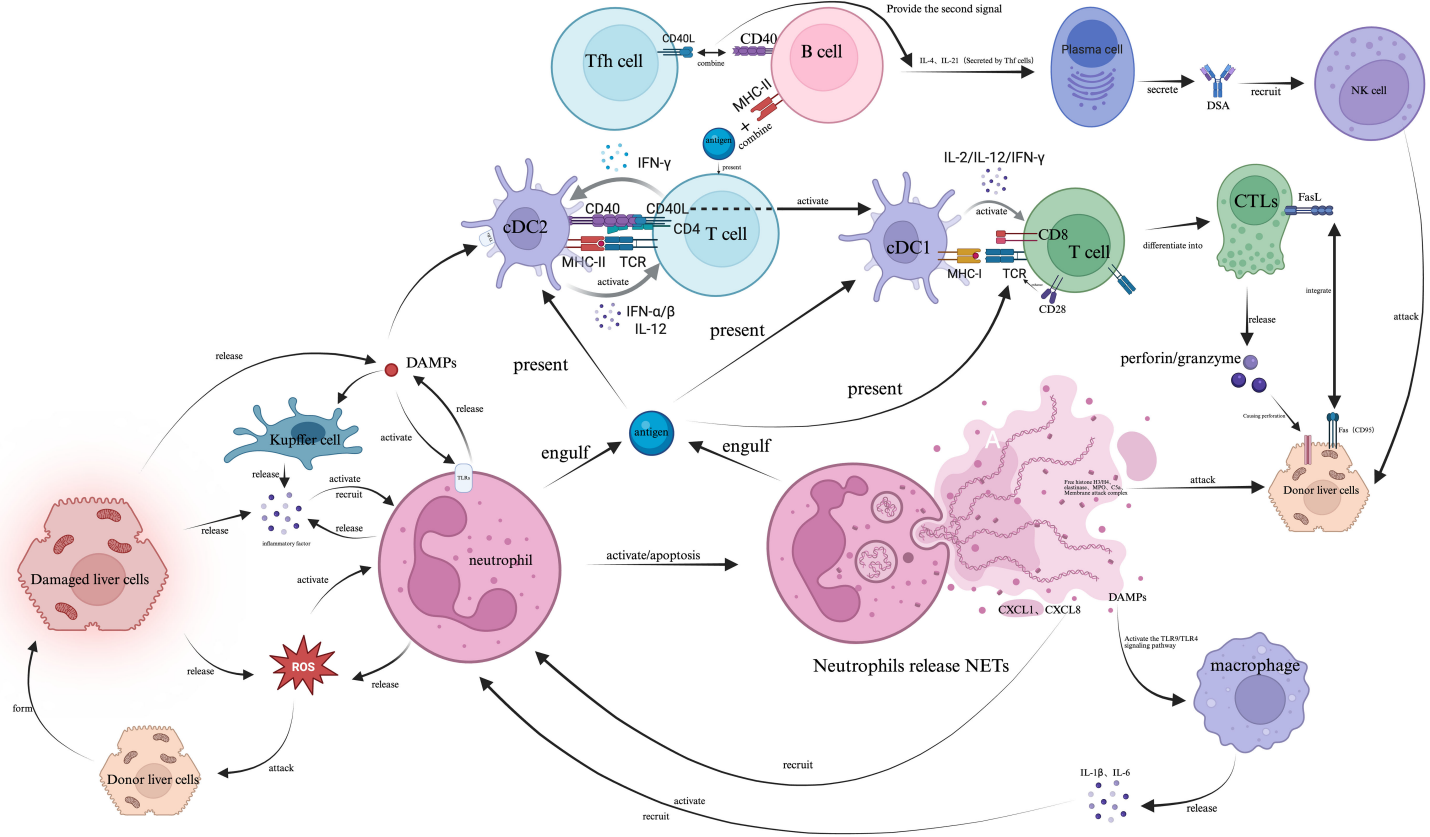
Graft liver ischemia-reperfusion injury triggers hepatocyte release of DAMPs (e.g., HMGB1, ATP), which activate Kupffer cells via TLR4 and other PRRs to secrete pro-inflammatory factors (ROS, IL-1*β*, IL-6) and recruit neutrophils. Neutrophils release NETs, causing direct tissue damage and amplifying inflammation by activating macrophages. This bridges to adaptive immunity where cDC2s present antigens via MHC-II to CD4^+^ T cells, which, with CD40-CD40L co-stimulation, differentiate into IFN-*γ* and IL-12-secreting Th1 cells. IFN-*γ* activates cDC1s to prime CD8^+^ T cells via MHC-I and CD28-B7, leading to CTL differentiation. Th1 cells also help B cells, via IL-4 and IL-21, to become DSA-producing plasma cells. The final effector phase involves CTLs killing graft cells through perforin/granzyme and FasL-Fas pathways, and DSAs mediating ADCC via NK cells, collectively causing graft rejection.

The physical migration of neutrophils into the hepatic parenchyma is a multi-step process orchestrated by adhesion molecules. Selectins play a pivotal and initiating role in the recruitment of neutrophils during LIRI (77). In the early phase of reperfusion, P-selectin and E-selectin expressed on activated endothelial cells and platelets interact with their primary ligand, P-selectin glycoprotein ligand-1 (PSGL-1), on circulating neutrophils (78, 79). This interaction mediates the critical initial step of leukocyte tethering and rolling along the vascular wall, This rolling process facilitates neutrophil activation by chemokines, which in turn triggers the conformational activation of integrins (80). Given their position at the apex of this inflammatory cascade, selectins represent a critical upstream therapeutic target to mitigate the extent of LIRI (78). Subsequently, chemokines like IL-8 and C5a activate GPCRs on neutrophils, triggering a conformational change in integrins. This leads to firm adhesion mediated primarily by Mac-1 (CD11b/CD18) and LFA-1 (CD11a/CD18) binding to ICAM-1 on endothelial cells, enabling neutrophils to resist shear stress and transmigrate into the liver tissue (28, 81, 82). Contrary to traditional notions, Monson et al. found that the cell adhesion molecules P-selectin and ICAM-1 do not appear to be crucial for neutrophil-mediated LIRI, whereas other cell adhesion molecules, such as VCAM-1, may be involved in the signal transduction of neutrophil extravasation (83) (**Figure 1**).

### 3.2 Clearance of neutrophils

Under physiological conditions, neutrophils undergo apoptosis and are promptly cleared by macrophages, during this apoptotic process, phosphatidylserine (PS) on the inner side of the neutrophil cell membrane flips to the outer side, which serves as an “eat me” signal (84, 85). M2 macrophages can recognize this signal through receptors such as CD36 and brain‐specific angiogenesis inhibitor 1 (BAI1) (86, 87), thereby phagocytosing apoptotic neutrophils and promoting the resolution of inflammation. However, this tightly regulated program is significantly disrupted during LIRI, leading to the accumulation of activated neutrophils and exacerbated tissue damage. The inflammatory microenvironment actively prolongs neutrophil survival through elevated levels of granulocyte-macrophage colony-stimulating factor (GM-CSF) and granulocyte colony-stimulating factor (G-CSF). These factors activate the JAK2/STAT3/STAT5 and PI3K/Akt pathways, resulting in the upregulation of anti-apoptotic proteins like Bcl-2 and Mcl-1 while inhibiting pro-apoptotic proteins, thereby delaying neutrophil apoptosis (88, 89).

During liver transplantation, activated neutrophils release NETs. These NETs act as a critical inflammatory amplifier, inducing the translocation and release of HMGB1 from the nucleus to the extracellular space. Once released, HMGB1 functions as a DAMP, binding to TLR4 and RAGE receptors on the surface of Kupffer cells. This interaction subsequently activates the MyD88/NF-*κ*B signaling pathway, driving the polarization of macrophages towards the pro-inflammatory M1 phenotype, which in turn results in a decrease in the phagocytosis of apoptotic neutrophils by macrophages. Ultimately, these M1-polarized macrophages release a cascade of pro-inflammatory cytokines, thereby exacerbating the immune-mediated injury and rejection of the transplanted liver (90, 91). Meanwhile, the reduction of anti - inflammatory cytokines such as IL-10 and TGF-*β* impedes the polarization process of pro - resolving M2 macrophages, thereby further weakening the clearance mechanism and ultimately resulting in the delayed apoptosis and aggregation of neutrophils (92).

Research has revealed an alternative clearance mechanism for neutrophils in models of sterile inflammation and kidney transplantation (93, 94). In this process, neutrophils reverse migrate from the injury site via JAM-C-dependent and LTB4-neutrophil elastase pathways, re-entering the circulation through lymphatic or vascular routes for eventual clearance in the bone marrow (95, 96). This non-destructive mechanism facilitates the rapid reduction of local inflammatory cells (97). However, it carries the potential risk of causing remote tissue injury. Although not yet extensively investigated in liver transplantation models, this pathway holds significant promise for mitigating the inflammatory response during the procedure.

### 3.3 Hepatocyte injury induced by neutrophils

During liver transplantation, the damage inflicted by neutrophils on hepatocytes is an intricate process that encompasses multiple molecular mechanisms (3). Once neutrophils infiltrate the liver parenchyma, they unleash their potent cytotoxic arsenal, inflicting multi - dimensional and synergistic lethal blows on hepatocytes. This attack process is primarily achieved through three major mechanisms: the oxidative burst driven by NADPH oxidase, the release of proteolytic enzymes via degranulation, and the formation of neutrophil extracellular traps with dual toxicity. These mechanisms do not act in isolation but rather interact and overlap, collectively constituting the core aspect of neutrophil - mediated hepatocyte injury (38).

Firstly, activated NADPH oxidase (NOX2) generates superoxide anion (O_2_^−^), which is converted to hydrogen peroxide (H_2_O_2_). MPO then utilizes H_2_O_2_ to produce highly cytotoxic hypochlorous acid (HOCl) (98). These ROS cause extensive damage, including lipid peroxidation of cell membranes, DNA strand breaks, and base modifications [e.g., 8-oxo-guanine (99)], leading to cells apoptosis or necrosis (100–102). Beyond direct oxidative damage, neutrophils can also exacerbate the inflammatory response and initiate specific cell death pathways. Reactive oxygen species directly activate the NLRP3 inflammasome, leading to the release of IL - 1*β* and IL - 18 (103). This release recruits more inflammatory cells, which in turn results in the production of reactive oxygen species and may form a vicious cycle of inflammation (104–106). Acting as pivotal secondary messengers, ROS orchestrate a multi-level amplification of the NF-*κ*B pro-inflammatory pathway (107). This process involves not only the oxidative activation of upstream kinases like TAK1 and IKK to hasten I*κ*B*α* degradation but also the oxidative inhibition of phosphatases to sustain the signal. Adding another layer of control, ROS directly enhance the transcriptional potency of the NF-*κ*B p65 subunit (108–110). Activated NF-*κ*B upregulates Fas expression on hepatocytes, priming them for apoptosis. Engagement with Fas ligand (FasL) from immune cells (e.g., CTLs, NK cells) triggers the death receptor pathway. This involves Death-inducing signaling complex formation and caspase cascade activation, leading to hepatocyte apoptosis and effectively converting inflammatory signals into a cell death command (111, 112). ROS can induce mitochondrial-dependent apoptosis by promoting cytochrome c release and caspase activation (113, 114).

Furthermore, neutrophils contribute to microcirculatory dysfunction and thrombosis. The deposition of NETs in hepatic sinusoids causes physical obstruction, reducing blood flow (115, 116). ROS-induced endothelial damage increases vascular permeability and upregulates adhesion molecules (ICAM-1, VCAM-1) and tissue factor, promoting a pro-thrombotic state. Platelet activation, further enhanced by ROS, exacerbates intravascular thrombus formation, worsening ischemia and hepatocyte injury (117).

**Figure 2:**
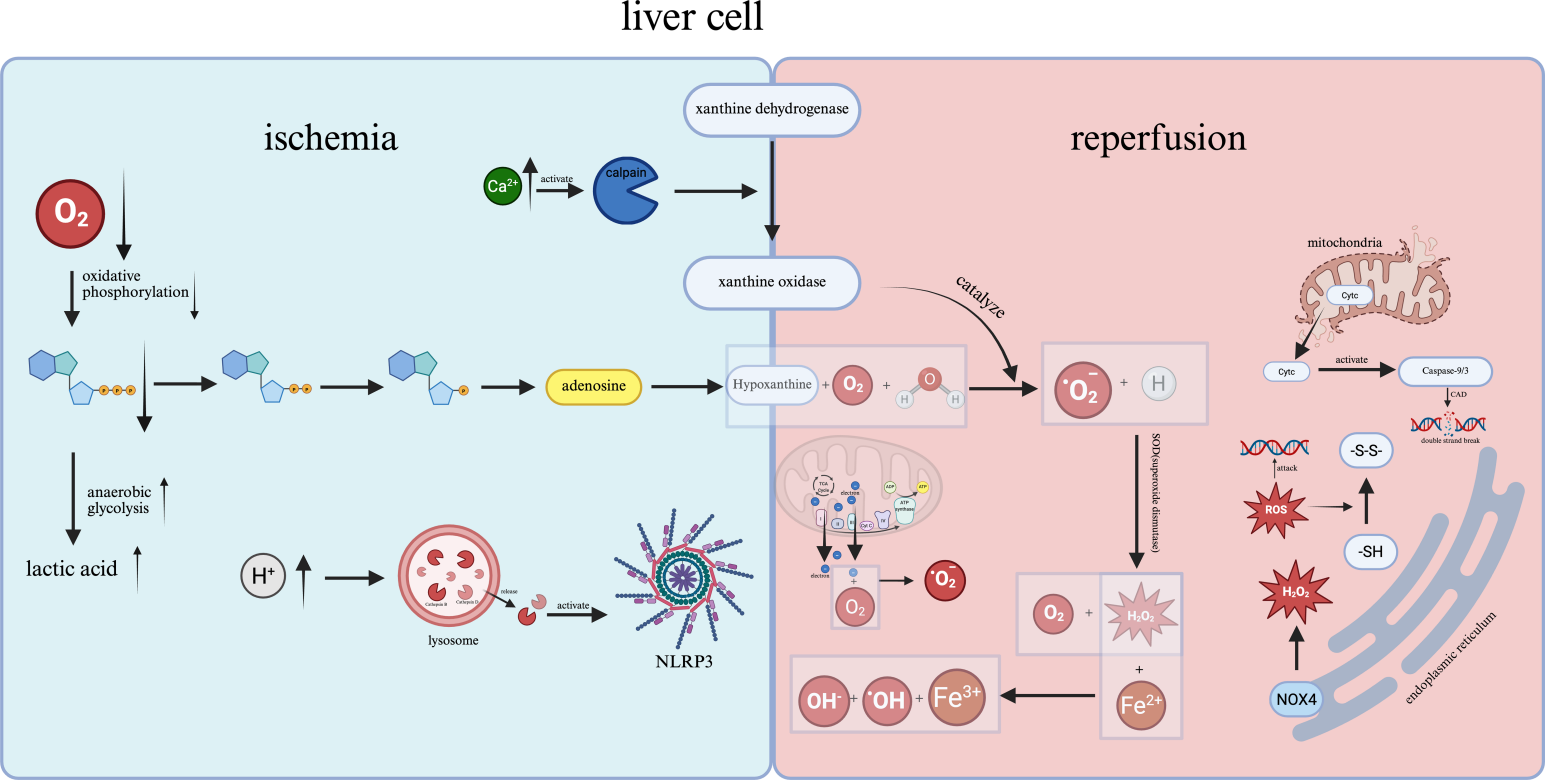
When hepatocytes enter the ischemic stage, hypoxia impairs oxidative phosphorylation and promotes anaerobic glycolysis, resulting in intracellular lactic acid accumulation. Under hypoxic conditions, calcium proteases and inflammasomes become activated, contributing to hepatic cell injury during the subsequent reperfusion phase. Additionally, the release of mitochondrial Cytc initiates the caspase cascade, ultimately leading to DNA fragmentation in hepatocytes.

In the inflammatory microenvironment associated with IRI following liver transplantation, neutrophils release neutrophil serine proteases (NSPs), including neutrophil elastase (NE), proteinase 3 (PR3), and cathepsin G, via degranulation and the formation of neutrophil extracellular traps (NETs). These enzymes function not only in a soluble form but also associate with exosomes to mediate intercellular signaling, thereby playing a pivotal role in matrix degradation, extracellular matrix remodeling, and fibrosis (38, 118). Upon activation, NSPs stored in azurophilic granules are released and possess broad-spectrum substrate specificity; they directly degrade ECM components such as collagen, elastin, fibronectin, and proteoglycans. This degradation exacerbates sinusoidal basement membrane disruption and increases sinusoidal permeability, promoting hepatocellular injury and microcirculatory dysfunction (119). Concurrently, by cleaving or activating cytokines, chemokines, and their receptors, NSPs reshape the local inflammatory milieu, indirectly disrupting the balance of ECM synthesis and degradation (120). Regarding pro-fibrotic mechanisms, EVs derived from inflammatory neutrophils carry active NE on their surface, which directly activates hepatic stellate cells (HSCs) via the ERK1/2 pathway. This activation promotes HSC proliferation, migration, and the deposition of collagen I and *α*-SMA, thereby driving pathological ECM accumulation in the context of pre-existing injury. Furthermore, NE and PR3 amplify intrahepatic inflammation under the regulation of the miR-223/STAT3 axis and synergize with TGF-*β* signaling to accelerate fibrosis progression (121, 122). Finally, NETs, characterized by a DNA scaffold decorated with granular proteins such as NE, PR3, and CG, exhibit both physical barrier and proteolytic activities. In complications including IRI, acute rejection, and thrombosis, NETs synergistically exacerbate ECM degradation and cellular injury while contributing to the evolution of late graft fibrosis by amplifying local inflammation and sustaining HSC activation (123) (**Figure 2**; **Table 2**).

**Table 2:** The roles of relevant cytokines during liver transplantation.

Factor/medium	Source/background	Effect on neutrophil	Key mechanisms	Reference
HMGB1	DAMP released from necrotic hepatocytes and Kupffer Cell	Potently activates, drives pro-inflammatory polarization	TLR2/4-MyD88, TRIF, RAGE → MAPK/IKK →NF-*κ*B/AP-1	(30, 42, 43, 70, 90)
Mitochondrial DNA	DAMP released from necrotic hepatocytes	Activates, induces pro-inflammatory cytokine production	TLR9/cGAS-STING	(44)
ATP	DAMP released from necrotic hepatocytes	Activates the inflammasome	P2X7/NLRP3	(45)
TNF-*α*, IL-1*β*, IL-6	Released during reperfusion by Kupffer cells, etc.	Further activates, enhances effector functions	Classic pro-inflammatory pathways like NF-*κ*B	(22, 74)
HIF-1*α*	Induced by hypoxia during the ischemic phase	Prolongs survival, inhibits apoptosis	Upregulates anti-apoptotic proteins (e.g., Mcl-1)	(39–41)
GM-CSF, G-CSF	Highly expressed in the inflammatory microenvironment	Delays apoptosis, extends lifespan	JAK2/STAT3/STAT5, PI3K/Akt → Bcl-2/Mcl-1↑; AKT-ERK1/2	(88, 89)
IL-10, TGF-*β*	During the inflammation resolution phase	Induces differentiation into N2 (anti-inflammatory/pro-repair) phenotype	IL-10R → JAK1/TYK2 → STAT3; TGF-*β*R → SMAD2/3/4	(14, 16, 124)
SPMs	Endogenous lipid mediators	Reprograms neutrophils into a pro-repair phenotype	NF-*κ*B inhibition; Activate PPAR-*γ*, STAT6, AMPK; Down-regulate Mcl-1	(57)
IL-1, CXCL2, CXCL8 (IL-8)	Secreted by damaged hepatocytes, endothelial cells	Establishes a chemotactic gradient, guides migration	CXCR1/2	(22, 28, 74, 81, 82)
C5a	Generated by complement system activation	Activates and enhances adhesion	GPCRs → Integrin activation	(28, 81, 82)
CXCL9/10	Secreted by pro-inflammatory neutrophils	Recruits effector T cells to the injury site	CXCL9/10-CXCR3	(33, 51)
BAFF	Secretion induced by NETs	Effect on neutrophils themselves is unclear	BAFF →B cells/Tfh cells↑→plasmocyte↑→DSA↑	(50)

## 4 Bridging innate and adaptive immunity in rejection reaction

### 4.1 Acute rejection

In the context of LIRI, neutrophil-mediated acute rejection (AR) primarily manifests through direct tissue damage, modulation of inflammatory responses, and synergistic interactions with other immune cells. Central to AR in liver transplantation are CD4 and CD8 T cell subsets, which orchestrate graft destruction via direct cytotoxicity, cytokine release, and the coordination of additional immune effectors (125). Neutrophils release a variety of inflammatory factors. These inflammatory factors interact with other immune cells, leading to the further development of the inflammatory response (5).

Neutrophils can function as non-professional antigen-presenting cells by upregulating MHC class II molecules, enabling them to present donor-derived peptides directly to CD4+ T cells and initiate the differentiation of effector subsets like Th1 and Th17 (126–128). In an IL-12-rich milieu, naïve CD4^+^ T cells become Th1 cells (129), which then unleash IFN-*γ* and TNF-*α* to powerfully activate macrophages and CD8^+^ T cells, orchestrating the cellular infiltration characteristic of acute cellular rejection (130–132). In parallel, a cocktail of IL-6, TGF-*β*, and IL-23 steers differentiation towards Th17 cells (133, 134). These cells, through IL-17 secretion, summon neutrophils to the graft, igniting a self-amplifying inflammatory loop that intensifies tissue injury (135). Moreover, the emergence of Tfh cells fuels the production of donor-specific antibodies (DSA), capable of triggering acute antibody-mediated rejection. Therefore, when the pro-inflammatory forces of Th1, Th17, and Tfh cells dominate and suppress the counter-regulatory influence of Tregs, the immune equilibrium is tipped decisively towards inflammation, culminating in the initiation and escalation of acute rejection (136). Furthermore, neutrophils can express co-2stimulatory molecules such as CD80, CD86, and OX40L, providing the essential “second signal” for full T-cell activation (137, 138).

Indirectly, neutrophils profoundly influence T-cell activation via dendritic cells (DCs). NETs and other neutrophil-derived DAMPs activate DCs through TLR9 and complement pathways, enhancing their maturation and antigen-presenting capacity (139). This synergistic interaction is supported by evidence that neutrophil depletion reduces T-cell infiltration and improves graft survival, and that neutrophils and T cells co-localize in rejecting liver biopsies (140).

### 4.2 Chronic rejection

In the field of liver transplantation, chronic rejection (CR) presents distinct pathological features. The principal pathological manifestations of CR following liver transplantation encompass the disappearance of bile ducts, arterial lesions, fibrosis, and a progressive decline in liver function. At present, it is widely acknowledged that CR is predominantly instigated by T cells and antibodies, and LIRI is one of the main risk factors for chronic rejection after liver transplantation (141–143). Clinical evidence links neutrophil activity to CR following liver transplantation. For instance, a significant early post-transplant elevation in the peripheral neutrophil-to-lymphocyte ratio (NLR) has been consistently identified as an independent risk factor for poor long-term graft survival and chronic graft dysfunction (144, 145). Furthermore, LIRI creates a persistent, pro - fibrotic microenvironment, which directly drives subsequent chronic rejection. Research has confirmed that an effective upstream intervention strategy is to activate the glucocorticoid receptor of hepatocytes through recombinant relaxin to alleviate acute ischemia - reperfusion injury. This strategy can fundamentally disrupt the pathological crosstalk from IRI to chronic rejection, thereby protecting the long - term function of the transplanted liver (35).

Notably, while numerous animal models have been developed to study AR, the number of models dedicated to CR remains limited. Given the complexity of the disease, more long-term investigations are essential to validate the applicability of these animal models to human conditions. In this context, the integration of single-cell sequencing and spatial transcriptomics holds great promise. This approach can be effectively employed to analyze the heterogeneity of neutrophil subpopulations in CR, thereby facilitating the identification and development of specific biomarkers. Such efforts are crucial for advancing our understanding of CR mechanisms and for the development of more targeted therapeutic strategies.

## 5 Intervention strategies targeting neutrophils

Elucidating the mechanisms by which neutrophils contribute to IRI, contemporary therapeutic approaches targeting these cells have centered on chemotaxis inhibition, regulation of NETs, ROS neutralization, and promotion of inflammatory resolution.

Neutrophils are recruited to the allograft liver via chemokine receptors CXCR1/CXCR2. Reparixin, an antagonist of this pathway, has been shown to diminish neutrophil infiltration, thereby alleviating IRI and early graft rejection. Its efficacy in mitigating inflammatory responses has been demonstrated in preclinical models (22).

Liraglutide activates the GLP-1R/AMPK signaling axis to shift macrophage polarization from a pro-inflammatory M1 to an anti-inflammatory M2 phenotype. This reprogramming, which involves inhibiting the NF-*κ*B pathway and activating the STAT6/PPAR*γ* pathways, is key to mitigating hepatic inflammation and injury (146). This indicates that liraglutide may serve as a potential drug for clinical treatment of LIRI.

It has been experimentally validated that curcumin, tetramethylpyrazine, and thrombomodulin confer significant hepatoprotection by inhibiting NETs formation. Regarding their mechanisms of action, curcumin and tetramethylpyrazine primarily mediate their protective effects through the direct suppression of NETosis (61, 147). Conversely, the hepatoprotective capacity of recombinant human thrombomodulin is attributable to its blockade of the TLR4 signaling cascade (148). Despite these promising results, the preclinical evidence is constrained by several pronounced limitations. Chief among these is the exclusive use of rat models, which poses inherent uncertainties for clinical translation. Moreover, the putative lack of target specificity for the natural compounds curcumin and tetramethylpyrazine leaves open the question of whether their observed efficacy is exclusively a consequence of NET inhibition. Finally, the intrinsic anticoagulant activity of thrombomodulin constitutes a potential confounding variable, which complicates the definitive attribution of its therapeutic benefit to NET suppression. Consequently, future investigations are imperative to corroborate these findings in models with greater human relevance and to delineate the precise molecular targets of these agents, thereby excluding alternative mechanistic explanations.

Targeting inflammation resolution with endogenous mediators like Ac2–26 and Resolvin D1 is a promising strategy against hepatic ischemia-reperfusion injury. Ac2–26 acts by disrupting the HMGB1/TLR4/NF-*κ*B inflammatory axis, while Resolvin D1 orchestrates tissue repair by modulating neutrophil activity and promoting pro-resolving macrophage functions. Nevertheless, their clinical advancement is impeded by preclinical evidence, poor pharmacokinetics, and incomplete mechanistic characterization (149, 150).

During the intricate pathophysiological cascade of LIRI subsequent to liver transplantation, EVs, encompassing exosomes, constitute a critical nexus for the dissemination and exacerbation of inflammation, thereby establishing an essential link to the orchestration of neutrophil-mediated responses (151). It has been elucidated that endogenous EVs, whose biogenesis is modulated by the IRF1-Rab27a signaling axis, act to potentiate hepatic injury, thereby representing a pivotal pathogenic mechanism (152). In stark contrast, exogenous EVs, most notably mesenchymal stromal cell-derived EVs (MSC-EVs), possess considerable therapeutic efficacy (153). The primary mechanism is predicated on the intercellular transfer of functional mitochondria to neutrophils, leading to the suppression of pathological NET formation and the consequent amelioration of inflammatory and histological damage (154). Additionally, the functional plasticity of EVs, evidenced by their concentration-dependent bioeffects, furnishes a strong rationale for leveraging EVs from normothermic machine perfusion (NMP) perfusate as potential biomarkers for graft quality evaluation and therapeutic stratification (155). Notwithstanding the immense promise, the clinical translation of these discoveries is beset by considerable challenges, encompassing the intrinsic heterogeneity of EV populations, the establishment of standardized large-scale manufacturing protocols, the precise determination of optimal dosing parameters, and the thorough investigation of their long-term safety profile within the intricate *in vivo* milieu.

It is important to note that the majority of these agents remain in experimental phases, necessitating further clinical validation for translation into practice. Excessive neutrophil suppression carries the risk of increased infection susceptibility, highlighting the critical importance of optimizing the timing and degree of intervention. Biological markers, including the neutrophil-to-lymphocyte ratio (NLR), along with insights obtained from single-cell gene sequencing and predictions generated by computational models, may serve as critical indicators for guiding treatment adjustments (156–160). Collectively, these strategies provide potential avenues for improving liver transplantation outcomes, particularly by attenuating IRI and mitigating early graft rejection (**Table 3**).

**Table 3:** Meta-analysis of treatment methods for ischemia-reperfusion and rejection reactions.

Drug	Effect on key mechanisms	The targeted disease	Experimental subject	Reference
Reparixin	Antagonizing CXCR1/2	IRI	WT and LysM-eGFP mice	(22)
Liraglutide	Activates the GLP-1R/AMPK signaling	IRI	C57BL/6J (B6) mice; GLP-1R^-/-^ mice	(146)
Tetramethylpyrazine	Inhibited formation of NETs though inhibition of NADPH oxidase	IRI	Male Sprague dawley rats	(61)
Curcumin	Inhibit the MEK/ERK pathway	IRI	C57BL/6 mice	(147)
Thrombomodulin	Block the TLR4 signal; Inhibit NETosis	IRI	Male Sprague dawley rats	(148)
Ac2-26	Disrupt the HMGB1/TLR4/NF-*κ*B axis	IRI	C57BL/6 mice	(149)
Resolvin D1	Enhancement of M2 via ALX/FPR2 activation; miR-146b/TRAF6/NF-*κ*B; PI3K/AKT	IRI	C57BL/6 mice; Sprague dawley rat	(161–163)
MSC-EVs	Inhibite inflammatory responses; Reduce oxidative stress responses; Suppress cell death	IRI	Male Sprague dawley rat	(153)
Sivelestat	Ninj1/CXCL1	IRI	Myeloid Ninj1-deficient mice	(164)
Lachnospiraceae	Pyruvate/FOXO3/ALOX15	IRI	MASLD rat	(165)
Anti-Properdin-Ab	C5aR; ROS; MAPK; CXCL-1/2	IRI	Male wild-type, B10D2nSn-Slc mice	(166)
MIG30	CXCL1/2/6	IRI	Mice; Fresh venous blood samples from healthy human	(167)
Diphenyleneiodonium	NADPH/ROS/PAD4; NETs	IRI; AR	Brown Norway rat; Lewis rat	(168)
Anti-*γδ*TcR antibody; Anti-IL17a antibody	*γδ*TcR; IL17a	IRI	C57BL/6 mice	(135)
Salidroside	HMGB1/TLR-4/MAPK	AR	Brown Norway rat; Lewis rat	(169)

## 6 Conclusions

Although our understanding of LIRI has advanced, neutrophils, which can constitute up to 70% of infiltrating immune cells within 6–24 hours of reperfusion, are still largely viewed as simple pro-inflammatory effector cells. However, this view is overly simplistic. Recent single-cell studies have revealed significant neutrophil heterogeneity, identifying distinct subsets with both pro-inflammatory and pro-repair functions, yet their dynamic regulatory mechanisms remain poorly understood. Future research must therefore prioritize multi-omic profiling to delineate these subsets and develop specific modulatory drugs. These efforts are clinically crucial, as HIRI is a major contributor to early allograft dysfunction, affecting up to 25% of liver transplants. For patients with compromised immunity, a more nuanced approach is needed to control neutrophil-driven inflammation without causing excessive immunosuppression.
